# Radiographic Progression in Patients with Rheumatoid Arthritis in Clinical Remission or Low Disease Activity: Results from a Swiss National Registry (SCQM)

**DOI:** 10.3390/jcm13237424

**Published:** 2024-12-05

**Authors:** Lena L. N. Brandt, Hendrik Schulze-Koops, Thomas Hügle, Michael J. Nissen, Johannes von Kempis, Ruediger B. Mueller

**Affiliations:** 1 Rheumazentrum Ostschweiz, 9000 St. Gallen, Switzerland; lena.brandt@fen-net.de; 2 Division of Rheumatology and Clinical Immunology, Department of Internal Medicine IV, Ludwig-Maximilians-University Munich, 80539 Munich, Germany; hendrik.schulze-koops@med.uni-muenchen.de; 3 Division of Rheumatology, University Hospital Lausanne (CHUV), University Lausanne, 1015 Lausanne, Switzerland; thomas.hugle@chuv.ch; 4 Rheumatology, Geneva University Hospital, 1205 Geneva, Switzerland; michaelnissen@gmail.com; 5 Clinic for Rheumatology, Kantonsspital St. Gallen, 9007 St. Gallen, Switzerland; johannes.vonkempis@kssg.ch

**Keywords:** rheumatoid arthritis, disease activity, radiographic progression, remission

## Abstract

**Background/Objectives:** The therapeutic aim for rheumatoid arthritis (RA) is to control disease activity and prevent radiographic progression. Various clinical scores are used to assess disease activity in RA patients. The DAS 28 score can define states of low disease activity (LDA) and remission. Despite achieving LDA or remission, radiographic progression may, nevertheless, occur. However, the rates and frequency of this occurrence have not been analyzed in detail. (1) To characterize radiographic progression in patients with persistent DAS 28-defined LDA or remission. (2) Analyze the potential benefits of modifying therapeutic strategies in response to observed radiographic progression in patients with persistent LDA or remission. **Methods:** An analysis was conducted on RA patients enrolled in the SCQM (Swiss Clinical Quality Management) cohort. Persistent LDA or remission was defined as DAS 28 ≤ 3.2 or <2.6, respectively, recorded at two consecutive follow-up time points. Inclusion criteria involved patients with a minimum of two sets of radiographs taken during these LDA and/or remission periods. Radiographic progression was measured using the Ratingen score, a numerical scale ranging from 0 to 190, which quantifies joint erosions. Repair was defined as a decrease in the Ratingen score > 5 points/year, while progression was characterized by an increase of >1, >2, or >5 points change in the Ratingen score within a one-year timeframe. **Results:** Among 10′141 RA patients, there were 1′447 episodes of remission and 2′614 episodes of LDA, with two sets of X-rays available for assessment during these episodes. The rates of radiographic progression (>5 points change in the Ratingen score per year) were 11.2% for LDA and 8.8% for remission. Therapeutic adaptations were made in 7.0% of patients in remission and 12.9% of patients in LDA following radiographic progression. After radiographic progression despite LDA, loss of LDA was observed in 19% of patients with treatment intensification versus in 8.5% under continued treatment during follow-up within 36 months. **Conclusions:** We report a considerable rate of radiographic progression occurring in RA patients with LDA or clinical remission. Notwithstanding minor radiographic progression, maintaining therapeutic continuity seemed more favorable than altering the therapeutic regimen.

## 1. Introduction

Rheumatoid Arthritis (RA) is a chronic inflammatory disease leading to joint damage [1]. Treatment decisions for RA patients, as recommended by the European League Against Rheumatism (EULAR), should consider disease activity, progression of structural damage, comorbidities, and safety concerns [2]. Conventional radiographs of the hands and feet are crucial for assessing radiographic damage progression, both in clinical trials and routine practice [3,4]. Over the past two decades, an encouraging effect on radiographic progression has been reported for various conventional, biological, and targeted synthetic DMARDs (disease-modifying anti-rheumatic drugs). The majority of clinical studies have shown inhibition or at least a deceleration in radiographic progression for all therapeutic compounds [5,6,7,8].

Disease activity can be measured by various scores, such as the Disability Assessment Score 28 (DAS 28) [9], Clinical/Simplified Disease Activity Index (C/SDAI) [10], and others not listed here. Radiographic progression is measured as a continuous variable, e.g., by the Sharp van der Heijde [11] or the Ratingen score [12], among many others. For pragmatic reasons, it can only be dichotomized into progression or non-progression. Clinical disease activity scores, continuous variables, can categorize patients as high, moderate, or low disease activity (LDA) or remission.

As defined by the EULAR recommendations, “inhibition of damage progression by radiography is still a pivotal outcome for the classification of a drug as a DMARD” [2,9,13]. However, little is known about the management approach when radiographic progression occurs despite clinically assessed LDA or remission. Several studies have shown that patients can experience radiographic disease progression despite achieving good clinical outcomes, such as low DAS 28 scores [14,15,16,17,18]. The question remains open whether radiographic progression is a good reason for a therapeutic change and whether treatment adaptions lead to a beneficial outcome considering the patient’s disease activity.

Hypothesis: this study analyzed whether changing therapy in patients with radiographic progression despite LDA or remission is beneficial.

## 2. Materials and Methods

### 2.1. Study Population

The analysis included all rheumatoid arthritis (RA) patients from the SCQM RA cohort who achieved a disease activity score (DAS 28)-defined low disease activity (LDA) (≤3.2) or remission (<2.6) designation at two consecutive follow-up visits, with a minimum interval of 90 days. Intervals of LDA or remission were defined between the first and the last documented time point in LDA or remission without any visits in between indicating loss of LDA or remission. Within intervals of LDA or remission, we searched for the first radiographic interval (≥2 sets of radiographs of hands and feet) to derive radiographic progression during LDA.

Radiographs were scored using the Ratingen score [12]. The Ratingen score is an erosion score ranging from 0 to 190, allowing the classification of joint destruction from 0 to 5 per joint. Each grade represents 20% of joint surface destruction. It is assessed in a total of 38 joints. The changes in Ratingen scores were standardized to describe yearly progression: Ratingen scores time difference between the radiographs in days ×365.25 days/year.

The patients/episodes were grouped according to their radiographical progression.

Repair: reduction of Ratingen score >5 points/year, no change: ±1, ±2, ±5 Ratingen points/year, progression: 5–10, 10–20, 20–40, >40 Ratingen points/year for patients in LDA or remission.

Demographic and clinical data including age, sex, time to diagnosis, rheumatoid factor positivity, anti-cyclic citrullinated peptide antibody (ACPA) positivity, body mass index, DAS 28, erythrocyte sedimentation rate (ESR), C-reactive protein (CRP), Health Assessment Questionnaire-Disability Index (HAQ-DI), and smoking status were analyzed for each patient group separately.

Clinical progression in relation to therapeutic continuity or adaptation was also assessed.

Patients in LDA or remission with radiographical progression were followed and observed for increases (number and time to increase) in DAS 28 ≥ 2.6 ≥ 3.2, grouped depending on their subsequent treatment scheme (adaptation vs. continuity approach).

The administered medicinal agents resulting in therapeutic change after radiographic progression in LDA or remission were analyzed independently for patients in LDA, remission, and radiographic progression. If a therapeutic change was introduced 20 days prior to 100 days after a radiographic progression, this patient was grouped as an intervention due to radiographic progression.

### 2.2. Statistical Analysis

The descriptive analyses of patient disease characteristics were compared using standard descriptive statistics. Continuous variables were compared using a Student’s *t*-test and categorical variables with a Chi^2^ (χ^2^) test. All statistical analyses were 2-sided at the 0.05 significance level. The analyses have been performed using GraphPad Prism 5 software and R.

Radiographic progression was analyzed as a continuous outcome (i.e., the yearly rate of damage progression).

Multivariate analysis was conducted, adjusting for potential confounders and including various clinical, radiological, and patient-centered functional scores. The confounders considered were age, sex, disease duration, rheumatoid factor and ACPA positivity, DAS 28 level, disease duration, and number of previously used DMARDs.

The baseline disease characteristics were compared using standard descriptive statistics. Ratingen scores, DAS 28, and HAQ-DI scores were analyzed with the Wilcoxon signed-rank tests.

## 3. Results

### 3.1. Group Definition

Out of 10′141 RA patients in the SCQM cohort (1998–2020), 5′525 patients were selected, with 6′962 episodes in LDA. Within these 6′962 episodes in LDA, 11′803 sets of hand and foot X-rays of hands and feet were taken, and 2′614 periods of LDA were available with ≥2 sets of radiographs to analyze radiographic progression during LDA.

Similarly, 4′051 episodes of remission in 4′051 RA patients were found. Within these episodes of remission, 9′020 sets of hands and feet X-rays were taken, and 1′447 periods in remission with ≥2 radiographs were available for analysis.

### 3.2. Definition of Radiographical Progression vs. Non-Progression

Radiographic non-progression was significantly more frequent among patients in remission compared to LDA. The radiographic progression was analyzed as a calculated yearly increase in Ratingen scores of >1, 2, or 5 points/year.

**Figure 1:**
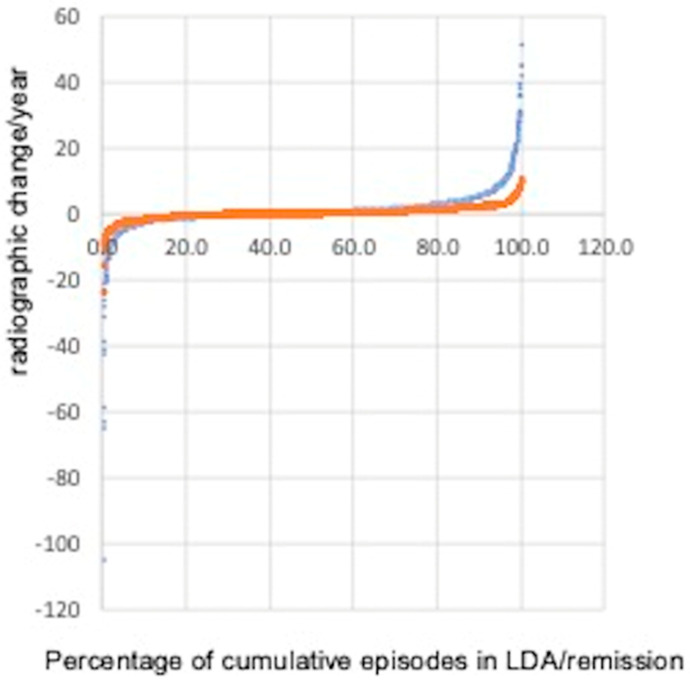
Radiographic-determined change/year within periods of LDA (blue) and remission (red) were analyzed separately for both patient groups. The groups were normalized to reflect progression observed between 2 sets of radiographs: 1447 patients in remission and 2614 patients in LDA.

In detail, 374 (25.8%), 146 (10.1%), and 128 (8.8%) patients progressed radiographically in remission, as compared to 1075 (38.0%), 739 (26.1%), and 317 (11.2%) in LDA with > 1 (χ^2^: 78.5139, *p* < 0.00001), 2 (χ^2^: 50.3102, *p* < 0.00001), or 5 points in the Ratingen score/year (χ^2^: 5.768, *p* = 0.016), respectively (Table 1, Figure 1).

In parallel, 208 (4.8%) patients in LDA and 23 (1.6%) patients in remission developed repair, as defined by a decrease in Ratingen scores > 5 points (Chi-square 286.7425, *p* < 0.00001, Table 1, Figure 1).

### 3.3. Demographic Data

Based on these definitions of radiographic progression in LDA or remission, demographic data were analyzed per group and for the respective subgroups: patient groups were similar for age, sex, BMI, disease duration, DAS 28, HAQ-DI, ACPA, and rheumatoid factor independently, whether their changes in Ratingen scores/year were judged as repair, status quo, or progression independently or whether the patients were in continued remission or LDA. Interestingly, patients developing repair of joint erosions were younger (59.0 years vs. 68.7 years, all patients in remission in the analysis) and less frequent rheumatoid factor positive (60.9 vs. 71.5%, all patients in remission in the analysis, Table 1 and Table 2).

### 3.4. Therapeutic Changes After Radiographic Progression

A total of 57 patients (7.0%) in remission and 105 patients (12.9%) in LDA underwent therapeutic changes within 90 days following the detection of radiographic progression (χ^2^: 4.203, *p* < 0.0002) (Table 3).

When we analyzed how much progression was required to result in therapeutic change, we found that 57.1% of patients in remission underwent a therapeutic change compared to none in LDA with a radiographic progression with a maximum of 1 Ratingen point/year (Table 3).

Conversely, 71.5% of patients in LDA with radiographic progression > 2 points in the Ratingen score/year underwent a therapeutic change compared to 19.6% of patients in remission (Table 3).

No differences in demographic data were found comparing patients staying in therapy as compared to changing therapy subsequent to radiographic progression in LDA and/or remission (Table 3).

The therapeutic strategies used after radiographic progression were oral glucocorticosteroids in 17.1% and 3.5%, conventional synthetic DMARDs in 46.7% and 35.1%, non-TNF biologic agents in 21.9% and 38.6%, TNF antagonists in 26.7% and 21.1%, and targeted synthetic DMARDs in 4.8% and 1.7% for patients progressing in LDA and remission, respectively (Table 3 and Table 4).

### 3.5. Clinical Follow-Up After Therapeutic Changes After Radiographic Progression Despite Remission

**Figure 2:**
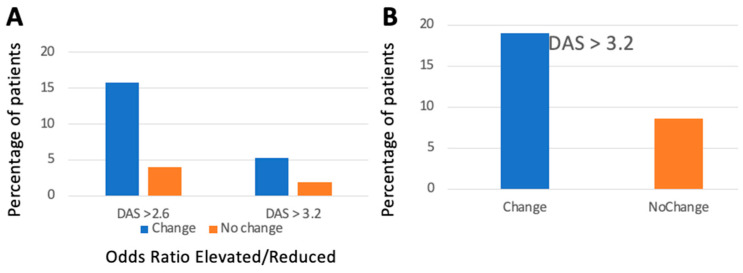
Frequency of radiographic progression: DAS 28 > 2.6 (left) and > 3.2 (right). (**A**) Remission, (**B**) LDA, Orange: No therapeutic regime change, Blue: Therapeutic regime change.

The frequency of patients who lost the status of a disease in remission was analyzed, revealing increases in DAS 28 > 2.6 in 15.8% and 4.1% (χ^2^: 5.8565, *p* = 0.016), and increases in DAS 28 > 3.2 in 5.3% and 1.9% (χ^2^: 0.5863, *p* = 0.44) during the next year of follow-up for patients with radiographic progression despite remission and changes in the therapeutic protocol, compared to patients who stayed on the same therapeutic protocol (Figure 2). The increase in DAS 28 > 2.6 occurred on average after 234.5 and 302.5 days (*p* = 0.06) for patients changing therapy or staying on the same therapeutic protocol, respectively. Likewise, the increase in DAS 28 > 3.2 occurred after 285.5 and 289.4 days (*p* = 0.95), respectively.

### 3.6. Clinical Development After Changing or Staying on Therapy Because of Radiographic Progression Despite LDA

The frequency of patients dropping out of LDA was analyzed, revealing rates of 19% and 8.5% (χ^2^: 11.0272, *p* = 0.0009) for increases in DAS 28 > 3.2 during the next year of follow-up for patients with radiographic progression despite LDA and changes in the therapeutic protocol, compared to patients who stayed on the same therapeutic protocol. The increase in DAS 28 > 3.2 occurred on average after 208.1 and 294.7 days (*p* = 0.06) for patients changing therapy or staying on the same therapeutic protocol, respectively.

## 4. Discussion

In summary, we have demonstrated that radiographic progression despite LDA/remission is common. It occurred more frequently in patients in LDA (38.0%) compared to those in remission (25.8%, Figure 1). Therapeutic changes following radiographic progression were infrequent. These changes were more common in patients with LDA (12.9%) than in those with remission (7.0%). When analyzing clinical disease activity after a therapeutic change due to radiographic progression despite LDA or remission, it more frequently worsened compared to patients who remained on the same therapeutic regimen.

The question of whether achieving remission or the lower rate of radiographic progression in patients with remission discouraged rheumatologists from changing therapy remains unanswered and cannot be addressed in this analysis. Surprisingly, smaller changes in Ratingen scores (≤1 point/year) were associated with a therapeutic change in patients in DAS 28-defined remission compared to patients in LDA. On the other hand, higher rates of radiographic progression (>2 points of Ratingen score) were more frequently associated with subsequent therapeutic changes.

Since the percentages of patients with radiographic progression did not differ based on the annual rate of progression when comparing patients with and without subsequent therapeutic changes, we reject the hypothesis that therapeutic change may be influenced by the degree of radiographic progression (Table 3). This rejection of the hypothesis is independent of the achieved clinical status of LDA or remission.

Therefore, whether the detection of smaller radiographic changes in patients in remission may lead to the urge to react to these minor changes remains open. However, our data indicate that patients may benefit if their treatment regime remains unaltered.

Secondly, there is no standardized strategy for how to react to radiographic progression despite LDA/remission (Table 4). Whether a specific strategy may help to prevent clinical progression after therapeutic change remains an open question.

We believe that the problem addressed is challenging to analyze in clinical trials or long-term follow-ups. Therefore, we hope that data from other registries may address the same question, giving us insight into the situation in other countries.

Thirdly, the data show less radiographical progression in remission compared to low disease activity. Thus, aiming for remission is beneficial in inhibiting radiographic progression as compared to LDA.

Interestingly, repair occurred more frequently in LDA than in remission (Figure 1). We hypothesize that minimal inflammation may be beneficial for stimulating the restructuring/repairing of damaged tissue. This hypothesis, suggesting that inflammation may promote repair, is also supported by other studies [19].

### Weaknesses

The analysis is based on a registry, and missing data, as with any registry, is a concern. It is conceivable that patients with complex acute issues are less likely to be documented in registries, as the focus is on stabilizing symptoms and not updating databases. It is also feasible that time constraints of medical professionals may also play an important role.

Secondly, we had a central scoring of radiographs, but they were not consecutively scored for all patients.

Like other scoring systems, the Ratingen score only focuses on radiographic destruction and not joint space narrowing. However, radiographic erosions are more accessible to detect than joint space narrowing. It is our believe that incorporating additional information from the Sharp van der Heijde score may not provide substantial benefit for clinical decision making.

A major issue of this paper is the definition of LDA and sustained remission. Other criteria like S/CDAI or Boolean-defined remission were not analyzed in this paper. Furthermore DAS 28 remission defined <2.6 has been discussed as not being a sufficient basis for defining remission [20,21,22].

Next, the definition of sustained remission as a minimum of three months for the data collection does not suit the requested standard of six months. On the other hand, no patients with sustained LDA/remission shorter than 6 months were included in the analysis.

## 5. Conclusions

In conclusion, we have demonstrated that radiographic progression despite LDA/remission is frequent. However, reacting to radiographic progression may not be necessary for the patients in LDA/remission.

## Figures and Tables

**Table 1:** Change in Ratingen scores in patients in LDA or remission.

	LDA (*n* = 2826)	Remission (*n* = 1447)
Repair *	4.8%	1.6%
No change ±1 Ratingen points/year	41.2% *	58.7% *
No change ±2 Ratingen points/year	61.0% *	81.8% *
No change ±5 Ratingen points/year	84.0% *	96.5% *
Progress 5–10 Ratingen points/year	7.0% *	1.9% *
Progress 10–20 Ratingen points/year	3.0% *	0.8% *
Progress >40 Ratingen points/year	0.14%	0%

* Statistically significant differences *p* < 0.05.

**Table 2:** Demographic data.

	LDA					Remission				
Change in Ratingen Score (Points/Year) (Years)	Repair > −5	No Change, ±1	No Change, ±2	No Change, ±5	Progress >5	Repair >−5	No Change, ±1	No Change, ±2	No Change, ±5	Progress >5
Age (years, av)	50.0	53.8	56.9	56.9	59.0	59.0	69.1	69.0	68.9	67.7
Sex (% female)	69.8	69.6	72.4	72.4	65.2	65.2	70.4	70.1	70.5	78.6
Time to diagnosis (years)	7.4	7.0	6.4	6.4	13.1	13.1	14.2	14.1	14.2	18.1
Rheumatoid factor pos. (%)	70.8	76.7	70.3	71.6	60.9	60.9	69.3	70.5	71.3	92.9
ACPA pos. (%)	57.9	66.3	43.4	40.8	73.7	73.7	70.3	70.8	71.3	84.2
BMI (kg/m^2^, av)	24.7	25.2	25.1	25.1	28.5	28.5	25.6	26.0	26.1	19.0
DAS 28	1.8	1.9	2.3	2.3	2.1	2.1	1.8	1.8	1.8	1.8
ESR (mm/h, av)	8.5	8.7	8.9	8.9	14.7	14.7	11.1	11.4	11.4	10.2
CRP (mg/L, av)	4.6	3.9	4.0	4.3	4.7	4.7	3.7	3.5	3.4	1.0
HAQ-DI	0.4	0.4	0.8	0.8	0.8	0.8	0.4	0.4	0.4	1.2
Smoking current (%)	12.3	12.6	20.8	23.3	22.2	22.2	16.6	15.4	14.7	14.3
Smoking ever (%)	26.9	25.9	29.2	28.8	22.2	22.2	43.6	42.3	41.7	14.3

For patients with double intervals with radiographic change and LDA, the first data entry was used for the analysis to avoid duplicates.

**Table 3:** Change in therapy after radiographic progression.

	LDA		Remission	
	Change Therapy (*n* = 105)	Stay on Therapy (*n* = 706)	Change Therapy (*n* = 57)	Stay on Therapy (*n* = 729)
Age (years)	57.0	56.9	69.1	68.1
Sex (% female)	72.2%	72.4%	77.2%	73.5%
Time to diagnosis (years)	10.2	6.4	15.6	14.3
Rheumatoid factor pos.	74.6%	70.3%	77.2%	77.0%
ACPA pos.	38.5%	43.4%	71.9%	54.6%
BMI (kg/m^2^)	25.5	25.1	28.6	25.9
DAS 28	2.3	2.3	1.9	1.8
HAQ-DI	1.0	0.8	0.5	0.5
Smoking current	20.5%	20.8%	19.3%	26.3%
Smoking ever	23.0%	29.2%	19.3%	26.3%
No change +1 Ratingen points	-	-	30 (57.1%)	383 (52.5%)
No change +2 Ratingen points	30 (28.0%)	274 (30.1%)	14 (25.0%)	213 (29.2%)
No change +5 Ratingen points	39 (36.4%)	320 (39.8%)	10 (15.6%)	112 (15.4%)
Progress >5 Ratingen points	38 (35.1%)	210 (25.2%)	3 (4%)	24 (3.3%)

**Table 4:** Therapeutic agents used after radiographic progression in LDA or remission.

		LDA (*n* = 105)	Remission (*n* = 57)
	Therapeutic Agent	Number	Number
	Prednisone	18 *	2 *
csDMARXDs/other drugs	Chloroquine	10 *	- *
	Cyclophosphamide	1	-
	Sulfasalazine	11 *	1 *
	Leflunomide	14	8
	Methotrexate	13	11
Biologics	Abatacept	8	2
	Ixekizumab	1	-
	Rituximab	8	10
	Tocilizumab	6	10
TNF antagonists	Adalimumab	9	4
	Etanercept	7	3
	Golimumab	2	1
	Infliximab	10	2
	Certolizumab	-	2
tsDMARDs	Baricitinib	2	1
	Tofacitinib	3	-

* Statistically significant differences *p* < 0.05.

## Data Availability

Study-related data can be made available from the SCQM Foundation according to the SCQM Rules of Research after the publication of all study-related research objectives. Researchers interested in further analyzing the data resulting from this study can contact the SCQM Foundation (scqm@hin.ch). Data can only be used for scientific research. SCQM is an ongoing, long-term registry with no end date for data collection and data provision. The original contributions presented in this study are included in the article; further inquiries can be directed to the corresponding authors.
